# Parallel Age-Related Cochlear Neural Degeneration and Cortical Gain Adaptation in Normal-Hearing Humans

**DOI:** 10.1523/JNEUROSCI.2037-25.2026

**Published:** 2026-04-09

**Authors:** Jonatan Märcher-Rørsted, Søren A. Fuglsang, Gerard Encina-Llamas, Sam Watson, Charlotte Sørensen, Hartwig R. Siebner, Torsten Dau, M. Charles Liberman, Jens Hjortkjær

**Affiliations:** ^1^Hearing Systems Section, Department of Health Technology, Technical University of Denmark, Kgs. Lyngby 2800, Denmark; ^2^Danish Research Centre for Magnetic Resonance, Department of Radiology and Nuclear Medicine, Copenhagen University Hospital – Amager and Hvidovre, Hvidovre 2650, Denmark; ^3^Copenhagen Hearing and Balance Center; Ear, Nose and Throat (ENT) & Audiology Clinic, Rigshospitalet, Copenhagen University Hospital, Copenhagen Ø 2100, Denmark; ^4^Faculty of Medicine, University of Vic – Central University of Catalonia (UVic-UCC), Vic 08500, Catalonia, Spain; ^5^Department of Neurology, Copenhagen University Hospital - Bispebjerg and Frederiksberg, Copenhagen NV 2400, Denmark; ^6^Department of Clinical Medicine, Faculty of Health and Medical Sciences, University of Copenhagen, Copenhagen N 2200, Denmark; ^7^Eaton-Peabody Laboratories, Massachusetts Eye and Ear, Boston, Massachusetts; ^8^Department of Otolaryngology-Head & Neck Surgery, Harvard Medical School, Boston, Massachusetts 02115

**Keywords:** aging, auditory cortex, auditory system, cochlea, electrophysiology, neurodegeneration

## Abstract

Accumulating evidence indicates that aging is associated with degeneration of neural components in the cochlea even before elevated hearing thresholds indicate hearing loss. Yet, it remains uncertain how such “hidden” hearing loss might shape brain responses to sound. Age-related cochlear decline has been associated with hyperactivity in central auditory pathways, but similar hyperactivity could also arise with age-related brain changes in inhibitory neurotransmission, regardless of peripheral status. Here, we collected an extensive physiological assay of cochlear neural health in an age-diverse cohort of human participants of both sexes (*N* = 105, ages 18–77). Despite clinically normal hearing, the assay indicated pronounced age-related cochlear neural degeneration, including reduced electrocochleographic responses to high-level clicks from the cochlear nerve (ABR wave I) as well as reduced brainstem frequency-following responses to 326 Hz tone carriers. ABR wave V did not show the same age-related reduction, indicating a response gain specific to transient stimulation between the cochlea and auditory brainstem. In the auditory cortex, aging was associated with enhanced transient evoked responses and diminished repetition suppression. Older adults showed pronounced N1-P2 components to individual sound onsets in regular tone sequences at faster repetition rates (2 Hz), where younger adults showed more steady-state-like potentials with little P2 deflection. However, these cortical functional changes were not significantly correlated with measures of cochlear neural degeneration. This suggests primary brain aging may be a significant contributor to auditory cortical hyperactivity and altered gain adaptation, progressing in parallel with peripheral neural degeneration.

## Significance Statement

Aging is associated with declines in auditory processing in both the ear and brain. Hyperresponsivity to sound within the auditory cortex has been regarded as a hallmark feature of sensory damage, and hyperactivity observed in older adults could be attributed to age-related cochlear neural degeneration. However, here we present data suggesting that cortical hyperactivity can progress in parallel with advancing cochlear neural degeneration in aging humans with clinically normal hearing. This indicates that peripheral deafferentation may not be the sole driver of central gain phenomena and suggests that primary aging can lead to similar adaptations in cortical sound processing.

## Introduction

As with all senses, hearing declines with age. In the inner ear, aging is associated with a progressive degeneration of cochlear nerve fibers and hair cells (Wu et al., 2019). In humans, ∼40% of cochlear nerve fibers have been lost by age 50, and this neural degeneration occurs before significant loss of the inner hair cells they innervate (Wu et al., 2019). While outer hair cell damage raises cochlear and behavioral thresholds, cochlear neural damage has minimal effect on audiometric thresholds until it is severe (Schuknecht and Woellner, 1955; Kujawa and Liberman, 2009; Chambers et al., 2016). Yet, the consequences of such primary cochlear neurodegeneration in central auditory circuits that mediate sound perception remain unclear.

Both sensory and neural damage in the cochlea is associated with plasticity in the central auditory system, including centrally downregulated inhibition to compensate for reduced peripheral input drive (Kotak et al., 2005; Sarro et al., 2008; Scholl and Wehr, 2008; Resnik and Polley, 2021). Decreased cortical inhibition following peripheral insults has been associated with cortical hyperresponsivity or reduced adaptive gain control of neural responses to sound (Qiu et al., 2000; Sun et al., 2009; de Villers-Sidani et al., 2010; Resnik and Polley, 2021). However, normal aging may also lead to a more global decline in inhibitory neurotransmission throughout the brain, producing similar functional consequences but arising from system-wide aging mechanisms rather than peripheral pathology (Gao et al., 2013; Rogalla and Hildebrandt, 2020; Zuppichini et al., 2024). Hyperactivity or suppression deficits are common consequences of aging, observed across sensory and motor systems (Schmolesky et al., 2000; Peinemann et al., 2001; Yu et al., 2006; Hermans et al., 2018), and primary aging effects could impact auditory processing in ways that resemble changes attributed to peripheral damage (Ibrahim and Llano, 2019). The same response phenotypes could thus emerge from either peripheral pathology or age-related central changes. While the effects of sensorineural peripheral damage can be dissociated from aging in animal models, they are typically conflated in human correlational studies. This makes it unclear how cochlear neural decline shapes human brain responses to sound and whether cortical readouts could potentially be used to assess hearing health. Conversely, it is unclear whether age-related changes in cortical sound-evoked responses observed in humans, such as hyperresponsivity or reduced adaptive control (Amenedo and Díaz, 1998; Harkrider et al., 2005; Sörös et al., 2009; Lister et al., 2011; Herrmann et al., 2018, 2019; Presacco et al., 2019), are driven by hidden peripheral etiology or by general brain aging (or a combination hereof).

This ambiguity becomes particularly pertinent in auditory aging where cochlear neural degeneration (CND) may progress silently before clinical hearing loss emerges. Synaptic connections between cochlear inner hair cells and their peripheral axons are vulnerable to aging and acoustic overexposure (Kujawa and Liberman, 2009; Sergeyenko et al., 2013), but even substantial deafferentation leaves little impact on hearing thresholds if sensory cells are intact (Schuknecht and Woellner, 1955; Kujawa and Liberman, 2009; Chambers et al., 2016). In animal models, age-related CND is seen well before damage to the outer hair cells and the associated elevation of thresholds (Sergeyenko et al., 2013). In humans, effects of age on cortical sound processing have been extensively researched by comparing young and older individuals with clinically normal hearing (Goodin et al., 1978; Picton et al., 1984; Amenedo and Díaz, 1998; Herrmann et al., 2019), but normal audiometry does not preclude cochlear neural decline. On the contrary, CND appears a near inevitable consequence of normal aging (Wu et al., 2019). Human studies exploring the neural or perceptual consequences of CND have met mixed results (Johannesen et al., 2019; Grant et al., 2020; Gómez-Álvarez et al., 2023; Garrett et al., 2025), possibly because effects of aging and peripheral decline progress simultaneously but along different trajectories. There is thus a need to capture central and peripheral aging effects in parallel.

Here, we simultaneously characterized peripheral and central sound-evoked neural responses in a cohort of aging humans with clinically normal hearing. We devised an extensive physiological test battery of cochlear neural health, comprising suprathreshold electrocochleographic responses from auditory nerve fibers, frequency-following responses (FFRs) from the auditory brainstem, and stapedial reflexes mediated by sound-evoked auditory nerve activity relayed via the auditory brainstem to motoneurons in the facial nerve. Response reductions across these measures indicated widespread CND with advancing age despite clinically normal hearing. Stimuli in the peripheral assay were designed to simultaneously probe inhibitory processing in the later cortical responses to the same sounds. In the cortex, hyperresponsivity and reduced response adaptation to repeated sound stimulation were observed as might be predicted from the presence of CND but was not correlated with it. Instead, consequences of primary brain aging may dominate individual variability in cortical sound-evoked responses in aging humans.

## Materials and Methods

### Participants

A total of 105 participants were recruited based on audiometric profiles and age from our audiological database in three age groups: young (≤25 years of age, *n* = 33, 13 male, mean age 22.1 ± 2.4), middle-aged (26–49 years of age, *n* = 42, 24 male, mean age 33.6 ± 6.5), and older (≥50 years of age, *n* = 30, 8 male, mean age 61.5 ± 7.3). All participants took part in an audiological examination on the day of experimentation. This included audiogram measurements using both ER-3 insert earphones at octave frequencies between 0.25 and 8 kHz and Sennheiser HDA200 earphones at extended high frequencies (9, 10, 11.25, 12, 14 and 16 kHz). Participants were considered normal hearing and included in the study if their audiometric thresholds were below 20 dB hearing level (HL) from 0.5 to 2 kHz and below 40 dB HL at 4 kHz (Carcagno and Plack, 2020). Four participants had thresholds between 20 and 40 dB at 4 kHz, and the remaining had thresholds below 20 dB HL. Listeners with asymmetric audiometric thresholds above 20 dB at any frequency between 0.25 and 2 kHz were excluded from participation. The better-hearing ear was identified based on thresholds and used as the test ear in subsequent tests. Outer- and middle-ear function was evaluated with otoscopy and wide-band tympanometry. A general screening by means of standardized questionnaires was performed to exclude subjects who suffered from neurological conditions or severe chronic tinnitus. All subjects provided written informed consent to participate. The experiment was approved by the Science Ethics Committee for the Capital region of Denmark (protocol H-16.036.391) and was conducted in accordance with the Declaration of Helsinki.

### Clinical hearing profiles

#### Transient evoked otoacoustic emissions

Transient evoked otoacoustic emissions (TEOAEs) to clicks were measured in the test ear using the Interacoustics Titan system and standardized protocols. Nonlinear clicks were presented at a peak-to-peak equivalent (ppe) sound pressure level (SPL) of 83 dB at a presentation rate of 80/s for 120 s (9,600 sweeps). OAE responses to the clicks were evaluated from 4–12.5 ms succeeding each click presentation. Recorded responses which exceeded a 55dB SPL noise floor level were rejected from further analysis. Fourier-transformed epochs were divided into 1 kHz wide nonoverlapping frequency bands with center frequencies from 1 to 5 kHz, resulting in five bands of equal bandwidth. TEOAEs were evaluated as the average sound power in each frequency band (dB SPL). Frequency-band averaged responses were considered significantly different from the noise floor if the TEOAE amplitude exceeded 6 dB signal-to-noise ratio (SNR).

#### Middle-ear muscle reflex

The middle-ear muscle reflex (MEMR) was measured using click-noise-click sequences presented to the test ear. The ear-canal sound pressure was measured before and after a 500 ms white noise elicitor (0.5–2 kHz) at levels of 75–100 dB SPL in steps of 5 dB. The probe click was presented at 100 dB ppeSPL. Growth-level functions were computed for each participant by taking the absolute value of the frequency response differences between the pre- and post-elicitor click responses, summed across all frequencies. A linear function was fitted to the growth of change of the absorbed power with increasing elicitor level. For each participant, the slope of the resulting linear fit, representing the individual linear growth function, was used in the statistical analysis. For 10 of the participants, it was not possible to correctly position the probe, resulting in missing data for these subjects.

#### Noise exposure questionnaires

Guest et al. (2017) proposed the Noise Exposure Structured Interview (NESI; Guest et al., 2017). The interview aims to identify noisy events across the lifespan of each participant, in either recreational or occupational settings. The accumulated noise exposure scores were summed across the two types, yielding a single measure of accumulated noise exposure for each participant. Additionally, participants self-evaluated their experience of temporary threshold shifts (TTS; Brungart et al., 2019) via the following question: “Have you ever experienced a temporary change in hearing (dullness or muffled sound) after noise exposure”. Scores were recorded on a scale from 1 to 7, ranging from Never (1) to Several times a day (7). It should be noted that noise exposure questionnaire data, relying on memory recall across life, are associated with high uncertainty and may require very large data samples for sufficient statistical power (Le Prell, 2019).

#### Self-reported hearing status questionnaire (SSQ)

As a measure of self-reported hearing status, we applied the 12-question version of the Speech, Spatial and Qualities of Hearing Scale questionnaire (SSQ12; Gatehouse and Noble, 2004; Noble et al., 2013). The questions address listeners' listening difficulties in everyday acoustic environments and are designed to identify self-reported listening difficulties in speech understanding, spatial separation abilities, and the perceived quality of acoustic stimuli in everyday situations. An average SSQ12 score per participant was computed by averaging the scores across the 12 questions.

#### Adaptive categorical loudness scaling (ACALOS)

Loudness perception was assessed using the adaptive categorical loudness scaling test (ACALOS; Brand and Hohmann, 2002). Participants were asked to rate the perceived loudness of a series of noise bursts on an 11-category perceptual scale ranging from “not heard” to “extremely loud”. The stimuli consisted of 500 ms, 1/3-octave band noises with center frequencies of 0.5, 1, 2, and 4 kHz, presented at levels ranging from −5 to 105 dB SPL. The presentation level was determined adaptively based on previous responses. Noise stimuli were presented to the test ear only using Sennheiser HD650 headphones. Loudness perception curves were fit between categorical units (CU) and the presentation level (dB SPL), as described in Oetting et al. (2014). The fitted loudness model was used to extract the slope of the loudness function as a measure of loudness growth.

#### Digit span

The participants performed a reversed digit span test (Blackburn and Benton, 1957). Subjects were asked to recall sequences of single digit numbers between 1 and 9 and report the sequence in reverse order. On subsequent trials, the listeners were presented with sequences of an increasing number of digits (2, 3, 4,…, 8). Digit stimuli were presented via Sennheiser HD650 headphones at a self-adjusted comfortable level.

### Electrophysiology

#### Data acquisition

Electroencephalography (EEG) data were recorded from frontotemporal 18 scalp electrodes with the BioSemi ActiveTwo system. Simultaneously, electrocochleography (ECochG) was recorded from inside the left and right ear canals using tiptrode electrodes (Etymotic Research ER3-26A). The tiptrodes formed part of electromagnetically shielded ER-3 insert earphones wrapped in gold foil. The gold foil was connected to the BioSemi amplifier using custom alligator cables, including an active circuit to optimize impedance. To ensure optimal positioning of the tiptrodes, otoscopy was performed before insertion. EEG and ECochG recordings were digitized at 16,384 Hz. Measurements were made in a double-walled acoustically and electromagnetically shielded booth in which participants were lying down and asked to relax.

#### Auditory brainstem responses

Transient evoked auditory brainstem responses (ABRs) were measured using 100 μs clicks, presented at 80 dB normal hearing level (nHL; 115.5 dB ppeSPL) at a presentation rate of 9 Hz. To discriminate the action potential (AP) component from the earlier presynaptic summating potential (SP), we also measured click responses at a presentation rate of 40 Hz, where the AP amplitudes (but not SPs) are reduced due to adaptation of neural components (Kiang, 1965; Liberman et al., 2016; Grant et al., 2020). The clicks were calibrated by first adjusting a 1 kHz tone to 115.5 dB SPL and then noting the peak-to-peak voltage (ppV) on a digital oscilloscope [reference equivalent threshold (RET) SPL; ISO 389-6. 2007]. The click stimuli were then adjusted in level, ensuring that the noted ppV of the click stimuli was equivalent to that of the 1 kHz tone. The calibration was performed for both polarities. The clicks were presented with alternating polarity, including a jitter of ±3 ms for both presentation rates. A total of 3,000 clicks in each polarity and for each rate (12,000 in total) were presented. Continuous ECochG recordings were segmented into 30 ms trials (from 10 ms preceding the onset of each click presentation to 20 ms after). Line noise (50 Hz) was removed using a comb notch filter centered ∼50 Hz and the two first harmonics (100 and 150 Hz). The data were bandpass filtered between 100 and 3 kHz using fourth-order Butterworth filters, demeaned, and rereferenced to three central-frontal midline electrodes (Cz, FCz, Fz). Response waveforms were evaluated as the electrical potential from the ipsilateral tiptrode to the average of the three scalp reference electrodes.

Analysis of the ABR waveforms was performed as follows. First, all click presentations were examined for artifacts, rejecting any epochs containing activity exceeding ±20 mV. On average, 0.35% ± 0.57% of the trials were rejected in the 9 Hz condition and 0.58% ± 1.31% in the 40 Hz condition. The remaining clean trials were then weighted by the inverse of their variance and averaged (John et al., 2001). For 12 of the participants, it was not possible to collect click-evoked responses due to technical difficulties during the recordings. For an additional 10 subjects, the 40 Hz condition was not recorded.

Averaged time-domain waveforms were computed by summing responses from both polarities ((C + R) / 2) and corrected for sound-delivery and tube delay (−1.1 ms). Averaged waveforms were baseline corrected by the mean response from *t* = −1 ms to *t* = 0 s relative to the delay-corrected *t* *=* 0 s point for both presentation-rate conditions (9 and 40 Hz). All averaged waveforms were then visually inspected. Responses that were clearly contaminated by stimulus artifacts or abnormalities after visual inspection were excluded from further analysis. This was the case for seven participants, leaving 86 participants with valid ABR data for further analysis (rejected: young = 4, middle-aged = 7, older = 8).

In the electrocochleogram, peak amplitudes and latencies corresponding to SP and AP of wave I, as well as wave V were identified. AP and wave V peaks were first identified using automated peak detection on the subject averaged 9 Hz ABR waveform. The SP was defined as the maximum voltage between 0 and 0.8 ms, AP was defined as the maximum voltage between 0.6 and 2.3 ms, and wave V as the maximum voltage between 4 and 7 ms. For AP and wave V, we also defined the trough following the peak. For APs, the trough of the peak was defined as the minimum of the waveform from the peak value to 15 samples (0.9 ms). The wave V trough was defined as the minimum of the waveform from the peak value to 20 samples (1.2 ms) after. Following automated peak detection, each waveform was visually inspected, and peak values were manually corrected if necessary. In addition, we quantified the ratio between the AP and wave V (I/V ratio) based on their respective peak amplitudes.

#### Frequency-following responses

FFR stimuli consisted of 326 Hz pure tones presented at a rate of 2 Hz (250 ms stimulation, 250 ms silence, 10 ms ramps, 85 dB SPL) for six consecutive presentations followed by 500 ms of silence. Each participant was presented with 486 tone presentations for each onset polarity of the stimulus. The polarity was kept constant in each condition (i.e., not true alternating). EEG data were bandpass filtered from 80 Hz to 3 kHz using windowed linear-phase finite impulse response (FIR) filters and rereferenced to the ipsilateral tiptrode. Continuous recordings were segmented into trials of 600 ms (from −100 to 500 ms relative to tone onset) and resampled to 4,096 Hz. Negative and positive polarity trials were subtracted from each other to enhance phase-dependent components in the response ((C − R) / 2). We note that this procedure can also enhance cochlear microphonics (CM) contributions, but these hair cell potentials have previously been estimated to be limited at the stimulation frequency used here (Temboury-Gutierrez et al., 2024). Polarity-subtracted trials exceeding 30 mV were rejected (0.003 ± 0.03% trials rejected). For four subjects, it was not possible to collect FFRs due to technical difficulties during recordings. Clean trials were weighted by the inverse of their variance and averaged per channel (John et al., 2001). The FFR magnitudes were then evaluated in the frequency domain. The spectral magnitude of the response at the carrier frequency (326 Hz) was identified and compared with the surrounding noise floor (± 20 Hz). The SNR was calculated by taking the power of the target magnitude bin and dividing it by the average of the surrounding frequency bins. Modulation sidebands evoked from the 2 Hz presentation rate, and corresponding harmonics (±2 Hz to ±10 Hz, in steps of 2 Hz), were excluded from the noise floor calculation. FFR responses were considered significantly different from noise if *p* < 0.01 (1%) in an *F* statistic test (Dobie and Wilson, 1996). Nonsignificant responses were excluded from the statistical analysis, which was the case for 19 subjects (young = 4, middle-aged = 10, older = 5).

#### Envelope-following responses

Band-limited (0.5–2 kHz) noise burst stimuli (300 ms long, 576 presentations) amplitude-modulated at 120 Hz were used to measure envelope-following responses (EFRs) at the modulation frequency. The continuous data were bandpass filtered from 80 Hz to 2 kHz, resampled to 4,096 Hz, and rereferenced to the average of the two mastoid channels (P9 and P10). The recordings were then segmented into trials of 700 ms, from 100 ms preceding the onset of each noise burst to 600 ms after the noise burst. Trials exceeding 50 mV between 0 and 500 ms were rejected (0.01 ± 0.04% trials rejected) and weighted by their inverse variance. To improve spectral resolution, clean trials (0–500 ms) were then reshaped into epochs of 2 s, by concatenating groups of four trials. Spectral EFR magnitudes to the modulation rate of the stimulus (120 Hz) were identified and compared with the surrounding noise floor, similar to the analysis for pure-tone FFRs. To avoid contaminating the SNR estimate with energy from the modulation sidebands corresponding to the interstimulus intervals (ISIs; 1.25–0.43 Hz) and respective harmonics, spectral energy in frequencies ±5 Hz surround the target frequency bin was not included in the noise floor. Line noise (100 Hz) was also not considered to be part of the noise floor. We then computed a SNR metric for each participant, which served as an outcome measure for further statistical analysis. Responses that were not significantly different from the surrounding noise floor (*F* test) were excluded from further analysis (young = 11, middle-aged = 18, older = 17).

#### Auditory evoked responses at varying interstimulus intervals

The modulated noise burst stimuli used to extract EFRs were presented at four different ISIs: 0.8, 1.3, 1.8, and 2.3 s. This allowed us to analyze cortical evoked potentials to the onset of the individual noise bursts as a function of ISI. ISI was varied by first presenting six consecutive 2.3 s ISIs, before proceeding to the next ISI (1.8 s). This was repeated until the ISI reached the lowest value (0.8 s). The ISI was then increased in a stepwise fashion, creating sweep-like ISI stimulus blocks of 48 trials (Fig. 5*A*), similar to Herrmann et al. (2019). A total of 144 trials was recorded for each ISI. The noise stimuli were presented at 75 dB SPL. Continuous data were rereferenced to the average of two mastoid channels (P9 and P10) and resampled to 4,096 Hz. For analysis of the cortical response, the data were bandpass filtered between 0.7 and 30 Hz using windowed linear-phase FIR filters. The filtered data were then segmented into trials of 600 ms from −100 to 500 ms relative to the stimulus onset. Individual trials exceeding ±80 mV were excluded from further analysis (0.22 ± 1.66% of trials rejected). For each ISI condition, we calculated the mean auditory evoked response (AEP) from −100 to 500 ms after stimulus onset. Individual averaged responses were then baseline corrected by subtracting the average voltage between −100 and 10 ms from the response. We then extracted N1, P2, and N2 peak amplitude components for each ISI condition per participant as the maximum or minimum voltage in the waveform within predefined windows (N1: 80–150 ms, P2: 150–250 ms, N2: 200–400 ms; Herrmann et al., 2019). We observed considerable variability in the exact timing of the component peaks and therefore identified peaks individually per subject and per ISI condition.

#### Cortical evoked responses to repeated tones

We extracted cortical responses evoked by the tone sequence used to measure FFRs. Epochs of six isochronous tones repeated at 2 Hz were first preprocessed from 5 s before to 7 s after the onset of the first tone, rereferenced to the average of two mastoid channels (P9 and P10), and resampled to 1,024 Hz. The data were bandpass filtered from 0.7 to 100 Hz using windowed linear-phase FIR filters. Epochs from 0 to 3.5 s relative to sequence onset were extracted, and epochs with values exceeding ±150 mV were rejected as artifactual (0.016 ± 0.09% of trials rejected). Clean trials were then weighted by their inverse variance and averaged per channel for time-domain visualization.

To quantify periodic activity phase-locked to the tone repetition rate, we calculated the intertrial phase coherence (ITPC) for each subject and each channel. To estimate ITPC, we first calculated the FFT of each electrode response across time from 0 to 3 s. The complex output *F*_k_(*f*) for trial *k* = 1, …, *N* was then used to calculate the ITPC per electrode *n*:

$$\mathrm{ITPC}(\;f,n)=\left|\frac{1}{N}\sum_{k=1}^{N}\;\frac{F_{k}(\;f,n)}{\left|F_{k}(\;f,n)\right|}\right|.$$

The ITPC values were then averaged over centrofrontal electrodes (FP1, F3, AFz, Fz, T7, C3, FC3, C4, Cz, FCz, T8, F4, Fp2, FC4; Herrmann et al., 2019). To quantify response periodicity, we computed the ITPC ratio (ITPC_Q_) between the ITPC value at the frequency of the presentation rate (*F*_0_ = 2 Hz) and mean ITPC value for the first nine harmonics (4–20 Hz in steps of 2 Hz; Irsik et al., 2021). To evaluate the evolution of peak amplitudes across the six-tone sequence, we identified P1, N1, P2, and N2 peaks within fixed time windows (P1: 45–65 ms, N1: 65–150 ms, P2: 150–250 ms, N2: 200–400 ms; Herrmann et al., 2019; Irsik et al., 2021). Due to the attenuation of the N1-P2 complex with repetition, we chose to estimate P1, N1, and P2 latencies based on tone one, i.e., the transient onset potential. Response adaptation for a given peak (e.g., P2_adapt_) was quantified as the difference between the peak amplitude of the first tone and the mean peak amplitude across tones 2–6.

### Statistical analysis

Statistical analyses were performed in R and MATLAB. To examine effects of age, we fitted linear models to the different outcome measures that included age, sex, and as low-frequency hearing thresholds as predictors (Y∼Age + Sex + PTA_lf_). Resulting *p* values were corrected for multiple comparisons using the false discovery rate (Benjamini and Hochberg, 1995). PTA_lf_ was included as a regressor to account for any potential subclinical differences in low-frequency thresholds. As an alternative control, we selected a subgroup of participants using a stricter audiometric thresholds criterion of ≤15 dB HL from 0.5 to 8 kHz at any given frequency (Fig. S1). There was no effect of age on low-frequency PTA in the subgroup (*t*_(2,74)_ = 1.907, *p* = 0.163), but high-frequency thresholds increase beyond the upper frequency defining the criterion (*t*_(2,74)_ = 7.014, *p* < 0.001). The same linear models were fit to this subset of data as for the full dataset. All source data are available in Dataset S1.

#### Correlation analyses

Spearman’s rho correlations were computed between pairs of measures that showed age-related effects. Statistical significance of each test was corrected for multiple comparisons with Bonferroni’s correction. To visualize shared and distinct sources of variability between measures, we performed principal component analysis (PCA). Missing data were imputed using Euclidian distance *k*-nearest neighbors, and PCA was then performed on the *z*-scored data.

## Results

### Peripheral hearing status

#### Hearing sensitivity

**Figure 1.**
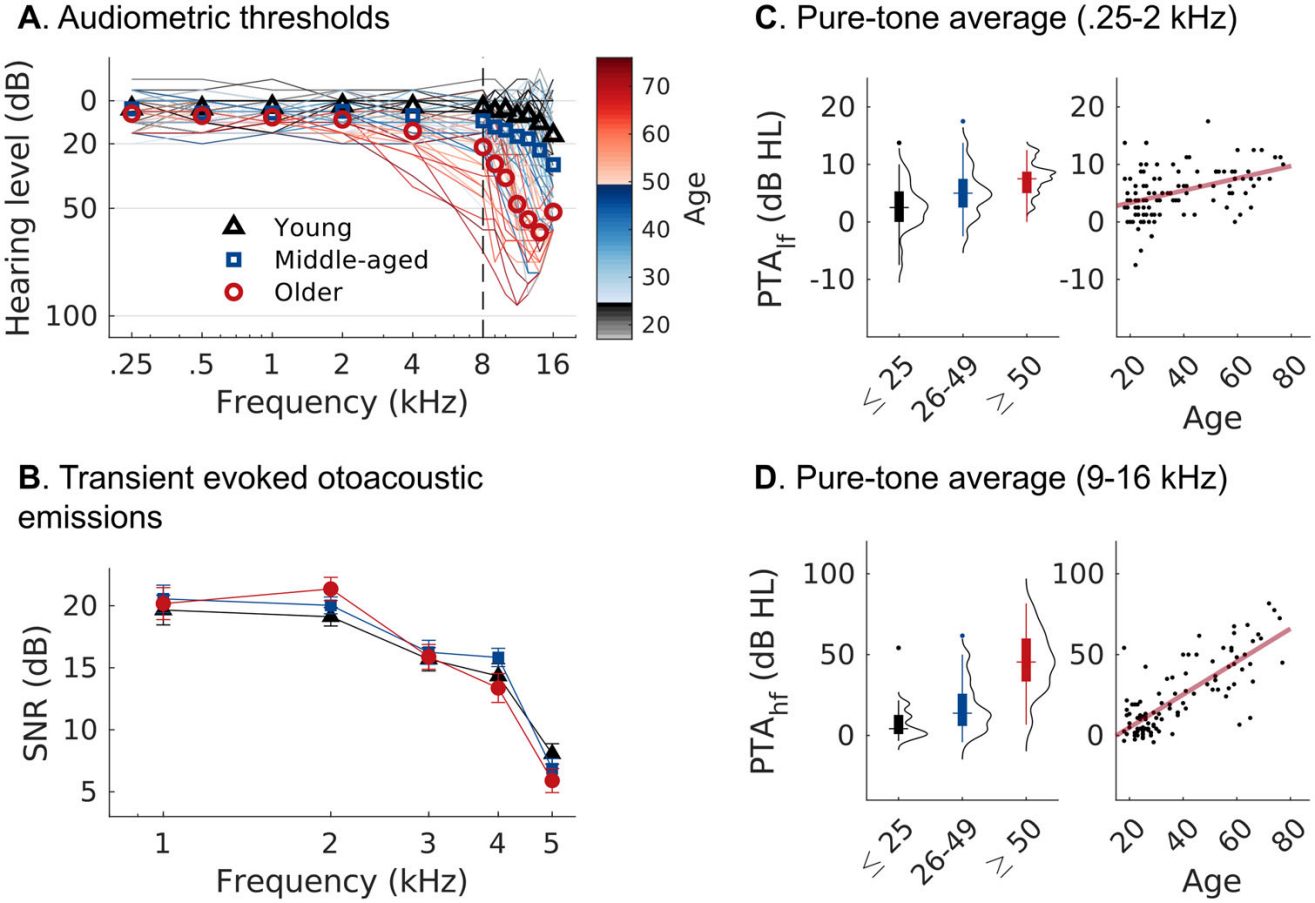
Hearing profiles of the healthy aging cohort. Black, Young (*n* = 33), age 18–25. Blue, Middle-aged (*n* = 42), age 26–49. Red, Older (*n* = 30): age 50–77. ***A***, Audiometric thresholds at lower frequencies indicate normal hearing, but thresholds at extended high frequencies (>8 kHz) were distinctly elevated in older participants. ***B***, No age-related differences in transient evoked OAEs at 1–5 kHz (± 0.5 kHz) indicating normal functioning mid-cochlear OHCs. ***C***, Low-frequency threshold averages (PTA_LF_: 0.25–2 kHz). ***D***, High-frequency threshold averages (PTA_HF_: 9–16 kHz).

Healthy participants (*n* = 105) representing a large age span (18–77), and with normal hearing as defined by audiometric thresholds, were recruited for the present study. Normal hearing thresholds at lower frequencies (i.e., 0.25–2.0 kHz) indicate relatively intact outer hair cells (OHCs), the main determinant of threshold sensitivity, in the apical half of the cochlea. As an objective correlate of OHC function, we measured transient evoked otoacoustic emissions (OAE) at 1–5 kHz (Fig. 1*B*) and found no differences across age groups. Although audiometry and OAE data indicate normal hearing across our cohort, extended high-frequency thresholds (9–16 kHz; Fig. 1*D*) were distinctly elevated with advancing age (PTA_HF_: *t*_(2,102)_ = 12.661, *p* < 0.001). Thresholds in the clinical lower frequency range (Fig. 1*C*) were also significantly correlated with age (PTA_LF_: *t*_(2,102)_ = 4.731, *p* < 0.001) despite being within the criterion for normal hearing for all participants. Such subclinical hearing decline is not unexpected given existing data on age-related loss of OHCs in human populations (Bredberg, 1968).

#### Auditory brainstem responses

**Figure 2.**
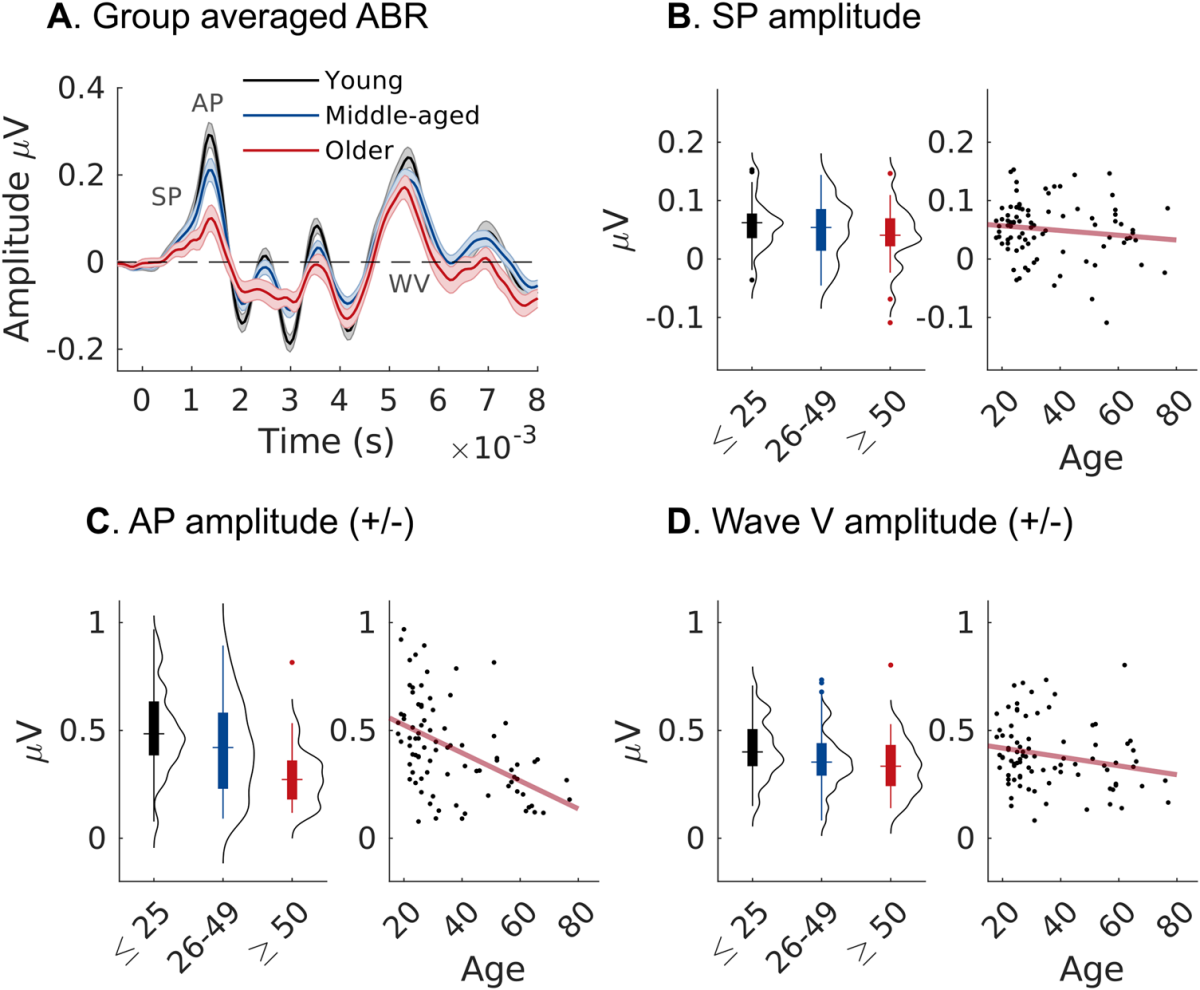
Auditory brainstem responses (ABRs) evoked by high-level clicks recorded from ear-canal electrodes. ***A***, Averaged ABR waveforms (±SEM) from young (black), middle-aged (blue), and older (red) participants. ***B***, Amplitudes of the SP (summating potential) component showed no effect of age. ***C***, Amplitudes of the action potentials (AP) component, representing summed spiking activity in the cochlear nerve, were reduced with increasing age. ***D***, Wave V, representing the click-evoked potential from the auditory midbrain, showed no age effect.

To assess cochlear neural function, we first recorded ABRs to high-level clicks from ear-canal electrodes (Fig. 2). Reduced suprathreshold amplitudes of ABR wave I, representing summed synchronous spiking activity of cochlear nerve fibers, is a primary functional measure of CND in animal models (Kujawa and Liberman, 2009). Here, we separated wave I of the ABR into the summating potential (SP), thought to be mainly generated by hair cells, and the action potential (AP) component generated mainly by cochlear neurons (Fig. 2*A*). AP amplitudes decreased significantly with increasing age (*t*_(3,82)_ = −5.370, *p* < 0.001; Fig. 2*C*), consistent with the presence of age-related CND in our normal hearing cohort. Age-related AP reductions were significant after controlling for effects of low-frequency threshold differences or when using a stricter clinical threshold criterion (≤15 dB below 8 kHz) to define a subset of participants without threshold differences (*t*_(2,63)_ = −3.736, *p* = 0.003; Fig. S1). AP amplitude reductions with age also remained significant after controlling for threshold differences at extended high frequencies (*t*_(3,82)_ = −3.249, *p* = 0.011). We found no effect of age on the SP component (*t*_(3,82)_ = −1.819, *p* = 0.179; Fig. 2*B*), consistent with the normal hair cell function across much of the cochlear spiral.

Wave V of the ABR (Fig. 2*A*) is dominated by the later click-evoked response in the inferior colliculus and associated areas of the auditory midbrain (Melcher and Kiang, 1996). We identified wave V as the fifth peak following the AP component (latency, 5.6 ms ± 0.3 ms) and found no significant age effect on wave V amplitudes (*t*_(3,82)_ = −1.800, *p* = 0.179; Fig. 2*D*). Thus, the age-related reduction in cochlear nerve response was not mirrored by response reductions in the auditory midbrain. We found no effect of age on the latencies of the AP (*t*_(3,82)_ = 0.204, *p* = 0.872) or wave V (*t*_(3,82)_ = 0.565, *p* = 0.682) peaks. The ratio of wave I:V also showed a significant effect of age (t_(3,82)_ = −4.219, *p* < 0.001).

#### Frequency-following responses

Although the suprathreshold wave I ABR amplitude indexes CND in animal models in which cochlear thresholds remain normal across all frequencies (Kujawa and Liberman, 2009), the relation can be confounded by high-frequency threshold shifts (and the basal-turn loss of hair cells they represent), as is often the case in human studies (Carcagno and Plack, 2020), including the present one. This has led to a pursuit of alternative physiological biomarkers of CND (Bharadwaj et al., 2019). We and others have proposed that the FFR phase-locked to the fine structure of a low-frequency high-level pure tone is sensitive to loss of cochlear nerve fibers across the cochlear spiral while being minimally influenced by basal OHC loss (Carcagno and Plack, 2020; Märcher-Rørsted et al., 2022; Temboury-Gutierrez et al., 2024).

**Figure 3.**
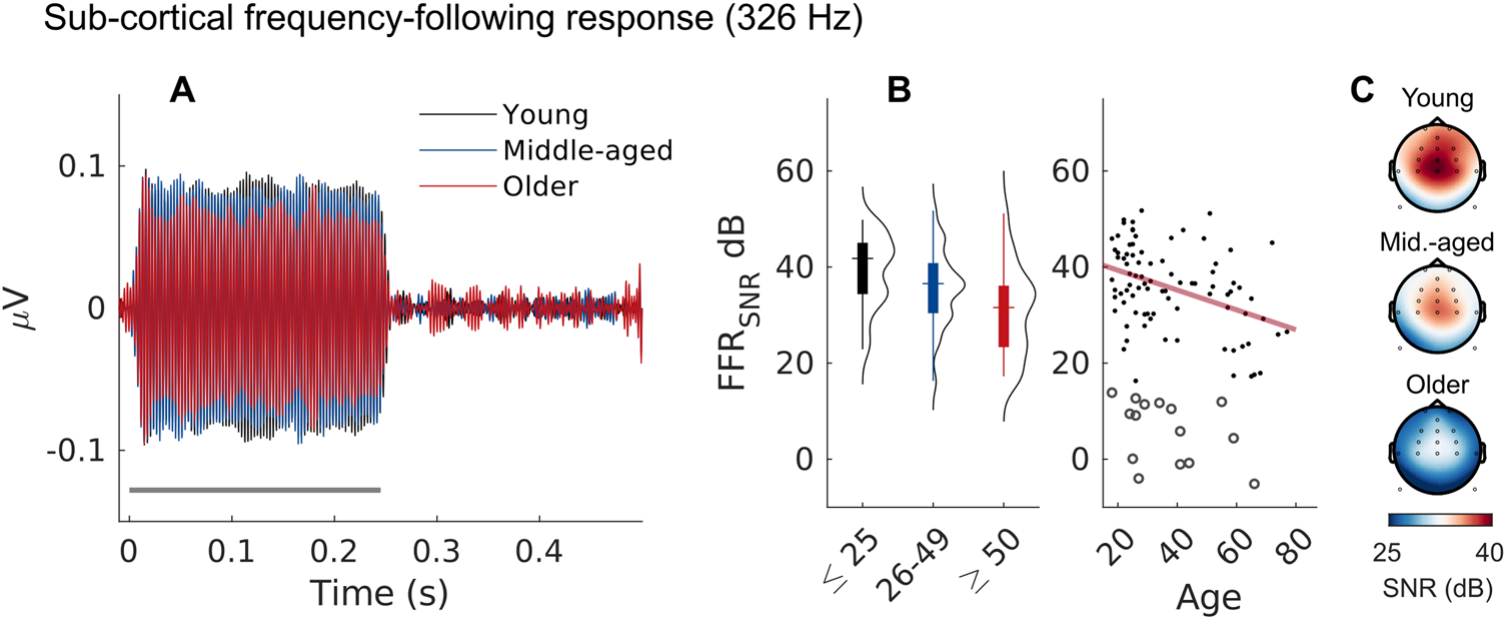
Frequency-following responses (FFRs) to a 326 Hz tone. ***A***, Averaged FFR waveforms showing phase-locked responses to the tone carrier. The solid line indicates the stimulation period (250 ms). ***B***, Reduced FFR_SNR_ with increasing age is consistent with cochlear neural degeneration. Circles indicate nonsignificant measurements. ***C***, Average magnitude of the FFR_SNR_ at central electrode positions in the three age groups.

We measured FFR responses to 326 Hz pure tones (85 dB SPL; Fig. 3). Figure 3*A* shows the time-domain FFR response phase-locked to the tone carrier for the different age groups. We evaluated the amount of phase-locked activity in the frequency domain to obtain a measure of signal-to-noise ratio (FFR_SNR_) in each participant. As expected, FFRs were significantly reduced with increasing age (FFR_SNR_: *t*_(3,82)_ = −4.360, *p* < 0.001; Fig. 3*B*), also when applying the stricter threshold criterion (*t*_(2,64)_ = −3.423, *p* = 0.005), again consistent with the age-related loss of cochlear nerve fibers (Märcher-Rørsted et al., 2022; Temboury-Gutierrez et al., 2024). The FFR_SNR_ was positively correlated with AP amplitudes (*ρ* = 0.377, *p* *<* 0.001, uncorrected), consistent with both measures being sensitive to age-related CND.

As an alternative CND index, brainstem responses following the envelope of amplitude-modulated stimuli (rather than the signal carrier) was initially considered in high-frequency hearing rodents (Parthasarathy et al., 2014; Shaheen et al., 2015; Parthasarathy and Kujawa, 2018) and later adapted to humans (Bharadwaj et al., 2015; Encina-Llamas et al., 2019; Grant et al., 2020; Vasilkov et al., 2021). Here, we measured EFRs to 120 Hz sinusoidally amplitude-modulated noise bursts. Unlike the FFR, however, significant EFR_SNR_ responses could only be obtained in a subset of 58 of 105 participants. This difference could relate to the difference in stimulus level (FFR: 85 dB SPL, EFR: 75 dB SPL) and the fact that we only considered single-polarity recordings leaving fewer trials for EFR measurement. For participants in which an EFR could be measured, we found no significant effect of age on the EFR_SNR_ after accounting for threshold differences (*t*_(3,55)_ = −1.601, *p* = 0.249). We found no significant correlation between the EFR and carrier FFR (*ρ* = 0.181, *p* = 0.203, uncorrected) or between the EFR and the AP amplitudes (*ρ* = 0.089, *p* = 0.536, uncorrected).

#### Middle-ear muscle reflexes

**Figure 4.**
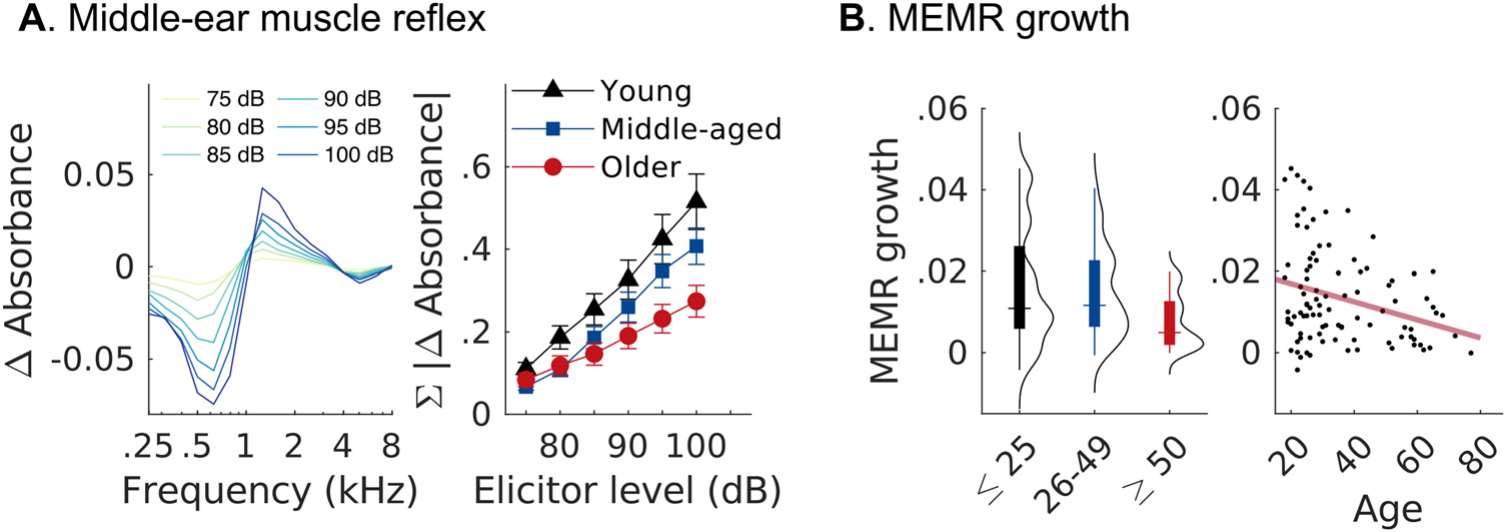
Middle-ear muscle reflexes (MEMRs) weaken with advancing age. MEMRs elicited by wide-band noise allow spectral averaging over a broad range of frequencies to index cochlear neural degeneration. ***A***, Left, Average MEMRs across participants for different elicitor levels. Stronger reflex activation causes a larger change in the absorbed sound power induced by the stiffening of the ossicular chain. Right, Average sum of absorbance changes as a function of elicitor level (±SEM). ***B***, MEMR level growth coefficients (75 to 100 dB SPL) decrease with advancing age.

The strength of the MEMR has been proposed as another measure of CND (Valero et al., 2016; Wojtczak et al., 2017; Mepani et al., 2019; Bharadwaj et al., 2022). Suprathreshold MEMR amplitudes are known to weaken with age (Mepani et al., 2019; Märcher-Rørsted et al., 2022) or auditory neuropathy (Berlin et al., 2005). Cochlear nerve fibers with low-spontaneous rate (SR) are thought to be important drivers of the MEMR (Kobler et al., 1992), and low-SR fibers may be particularly vulnerable to aging (Schmiedt et al., 1996). If this assumption is true, MEMRs should also be reduced in older listeners with clinically normal audiograms. Here, we measured MEMRs as the change in ear-canal sound pressure caused by the MEMR-mediated stiffening of the ossicular chain after presentation of a wide-band noise elicitor (Fig. 4*A*,*B*). We observed a main effect of age on the slope of the MEMR amplitude-versus-level function (*t*_(3,91)_ = −2.478, *p* = 0.049), suggesting shallower growth with increasing age. We found a significant correlation between the MEMR slope and AP amplitudes (*ρ* = 0.251, *p* = 0.024, uncorrected), as well as between the MEMR slope and carrier FFR_SNR_ (*ρ* = 0.244, *p* = 0.029, uncorrected) as expected from the fact that all these measures should depend on the level of sound-evoked activity in the cochlear nerve.

#### Behavioral audiological screening

Since overexposure to intense noise can induce CND in animal models without causing permanent threshold elevations (Kujawa and Liberman, 2009), past work has correlated physiological assays of cochlear function in humans with noise exposure estimates assessed by questionnaires. However, accumulated noise exposure across life is difficult to quantify (Le Prell, 2019), and this type of data is associated with high variability. For completeness, participants were screened with established questionnaires to assess life-time noise exposure (NESI), frequency of exposure to events causing temporary threshold shifts (TTS), as well as subjective hearing abilities (SSQ12). No effects of age were observed on any metric (Fig. S2*A–C*). To assess potential age-related change in loudness perception (Jahn et al., 2022), we administered the ACALOS test of growth in loudness perception with increasing levels of band-limited noise stimuli but observed no effect of age on the slope of the loudness perception curves (Fig. S2*D–F*).

### Cortical evoked responses

#### Amplified cortical transient responses

**Figure 5.**
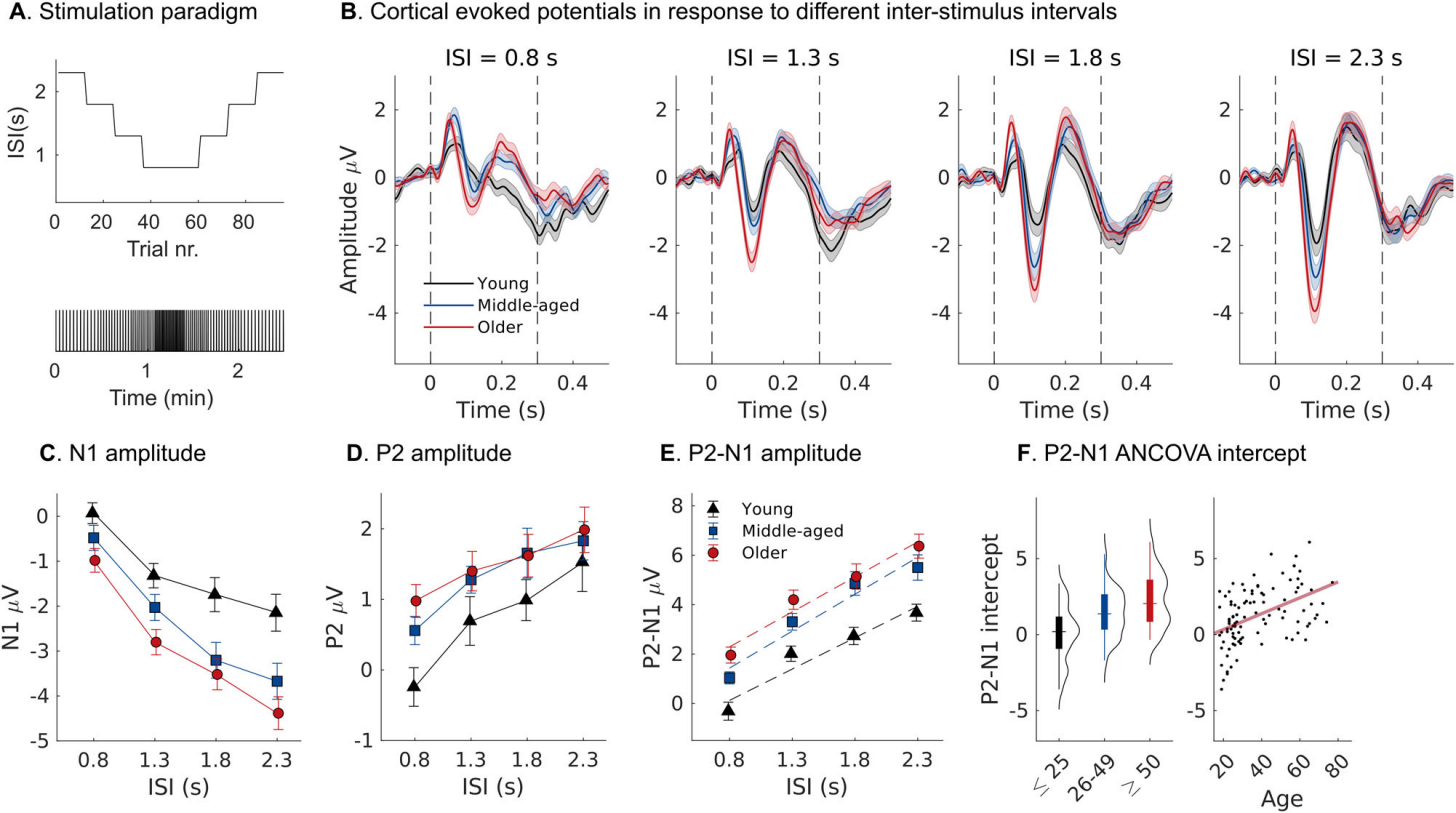
Amplified cortical AEPs evoked by noise bursts at varying interstimulus intervals (ISIs). ***A***, Stimulation paradigm: isochronous sequences of amplitude-modulated noise bursts (120 Hz, 300 ms) varied gradually in ISI between 0.8 and 2.3 s. ***B***, Baseline corrected group averaged AEP waveforms (±SEM) for the different ISIs. Dashed lines show stimulation onset and offset. ***C***, ***D***, Estimated N1 and P2 peak amplitudes as a function of ISI for different age groups. The P2 peak is preserved at the lowest ISI (0.8 s) in the older group. ***E***, ***F***, P2-N1 amplitude differences as a function of ISI. Group averaged linear model fits (dashed lines) show an intercept offset indicating a general response enhancement across ISI with increasing age.

The noise bursts used in our physiological assay were presented in isochronous sequences of varying ISIs (0.8, 1.3, 1.8 and 2.3 s) as illustrated in Figure 5*A*. We analyzed the scalp EEG for cortical AEPs evoked by the individual noise bursts within the sequence. Figure 5*B* shows group averaged AEPs at the four different repetition rates. At larger ISIs, the common transient AEP components with P1-N1-P2 deflections are seen (mean latencies: P1: 0.055 ± 0.007 s; N1: 0.114 ± 0.015 s; P2: 0.223 ± 0.026 s). In contrast to peripheral neural measures, cortical AEP amplitudes increased with advancing age. Larger N1 amplitudes in older participants were prominent at larger ISIs (ISI = 2.3 s: *ρ* = −0.373, *p* < 0.001, uncorrected). To quantify age effects across ISI, we computed linear growth functions for the P2-N1 peak amplitude difference (Fig. 5*E*). We found a significant association between the intercept of the P2-N1 growth (P2-N1_int_) and participant age (*t*_(3,96)_ = 4.050, *p* < 0.001; Fig. 5*F*), but no effect on the slope (*t*_(3,96)_ = 0.937, *p* = 0.507), indicating a general response enhancement across ISIs with increasing age.

The P2 component additionally showed a distinct age-related pattern of amplitude decrease with decreasing ISI (Fig. 5*D*). In young participants, the P2 disappears and gives way to a broader negativity at the lowest ISI (0.8 s), possibly indicating a transition toward steady-state activity at faster repetition rates (Holt and Özdamar, 2016), but this transition was not apparent in older participants. Instead, the P2 component of the transient response persisted at lower ISIs in older participants, leading to larger P2 amplitudes in older listeners at the shortest ISI (*ρ* = 0.333, *p* < 0.001, uncorrected).

#### Reduced cortical adaptation to tone repetition

**Figure 6.**
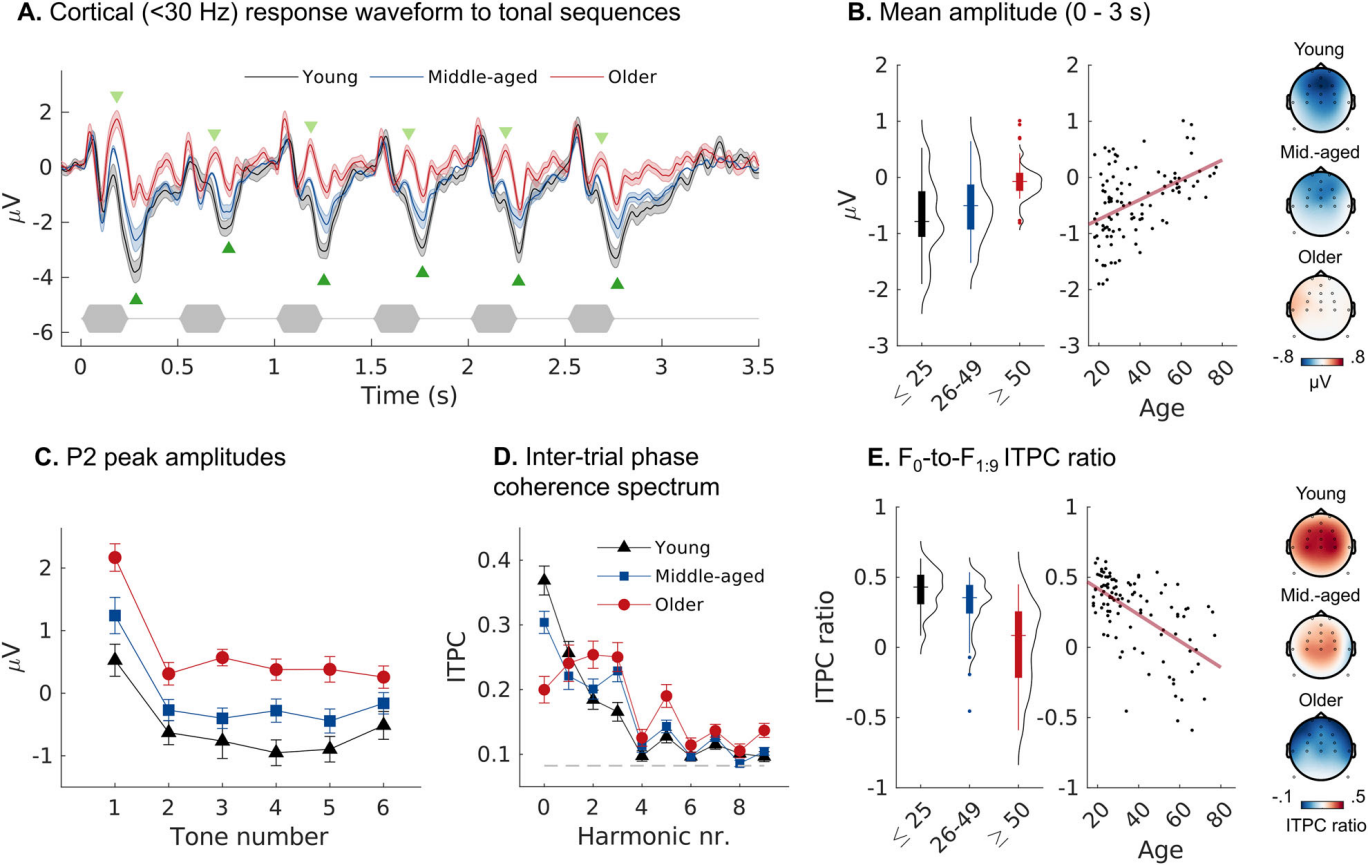
Cortical adaptive effects of tone repetition. Cortical AEPs to repeated pure tones (ISI: 0.5 s) show persistent transient onset responses in older participants. ***A***, Averaged response waveforms (±SEM) showing location of the P2 and N2 peaks (green triangles). The tone stimulation sequence is indicated below the response waveforms. ***B***, A significant age-related increase in mean response amplitude across the stimulation period is driven by the large mean negativity dominating the response waveforms in younger participants. ***C***, The P2 peak of the transient onset response persists with tone repetition in older participants. ***D–E***, ITPC spectral analysis of steady-state activity following the 2 Hz (*F*_0_) sequence repetition rate. Panel ***D*** shows the spectrum of harmonics from 2 Hz (*F*_0_) to 20 Hz (*F*_9_) for each age group. The dashed line indicates the noise floor. ***E***, The *F*_0_-to-*F*_1:9_ ITPC ratio decreases with advancing age indicating cortical responses increasingly dominated by response transients.

The age-related changes in AEP morphology observed at short ISIs (Fig. 5) might indicate suppression deficits or a reduced adaptive control of responses to repetitive signals. To further investigate this, we analyzed cortical AEPs evoked by the tone sequences used to measure the FFR. In this paradigm, stimuli were presented in sequences of six consecutive tones at a lower ISI of 0.5 s followed by a silent gap of 0.75 s (Fig. 6*A*) to analyze potential adaptive effects of sound repetition.

Figure 6*A* shows the waveforms of the cortical AEPs evoked by the six-tone sequence. A similar effect emerged as the one observed at short noise burst ISIs (Fig. 5). For older participants, transient AEPs with clear N1-P2 morphology were evoked by each tone in the sequence (Fig. 6*A*). In contrast, AEPs in younger participants had only small P2 deflections and instead a large broader N2-like negativity. Consequently, the mean negativity of the response (AEP_m_) computed across the stimulation period (0–3 s) decreased significantly with age (*t*_(3,99)_ = 4.784, *p* < 0.001; Fig. 6*C*).

The initial tone in the sequence evoked larger transient components compared with later ones (Fig. S3) suggesting adaptation or habituation to the repeating sequence. However, this response adaptation evolved differently with age. We found an interaction between age and tone repetition (first vs subsequent tones; Fig. S3) specifically for the P2 peak amplitudes (P2_adapt_: *t*_(4,202)_ = −2.317, *p* = 0.020). Whereas older participants showed persistent P2s throughout the sequence, the P2 became virtually absent after the initial tone presentation in the younger participants (Fig. 6*C*). Peak latencies of the different AEP components remained stable with repetition, with the notable exception of the N2 peak (Fig. S4). In young participants, N2 latencies decreased after sequence onset to stabilize ∼0.26 s (± 0.025 s), i.e., approximately half of the repetition period. This could indicate that the initial N2 transient evoked component is replaced by a different larger negativity that is part of steady-state activity following the sequence periodicity. This suspicion is supported by a stable longer N2 latency of 0.35 s (± 0.05 s) at longer ISIs in the noise-evoked transient responses reported above (Fig. 5). In older participants, however, N2 latencies decreased only gradually with tone repetition (Fig. S4), indicating a slower adaptation to the sequence periodicity with advancing age.

To capture these differences in the periodic nature of the response, we computed a spectral estimate of the response via the intertrial phase coherence (ITPC; Fig. 6*D*,*E*; Irsik et al., 2021). Older participants showed lower ITPC at the frequency of the presentation rate (*F*_0_: 2 Hz) but higher ITPC at *F*_0_ harmonics. In effect, the Q-ratio between the ITPC (ITPC_Q_) at the fundamental (*F*_0_) and its harmonics [log_10_(F_0_/F_1:9_); Irsik et al., 2021] decreased strongly with age (*t*_(3,99)_ = −6.409, *p* < 0.001; Fig. 6*E*). This again supports that cortical responses in young brains are dominated by activity following the sequence periodicity but become increasingly dominated by response transients evoked by individual sound onsets with advancing age.

#### Correlational analyses

**Figure 7.**
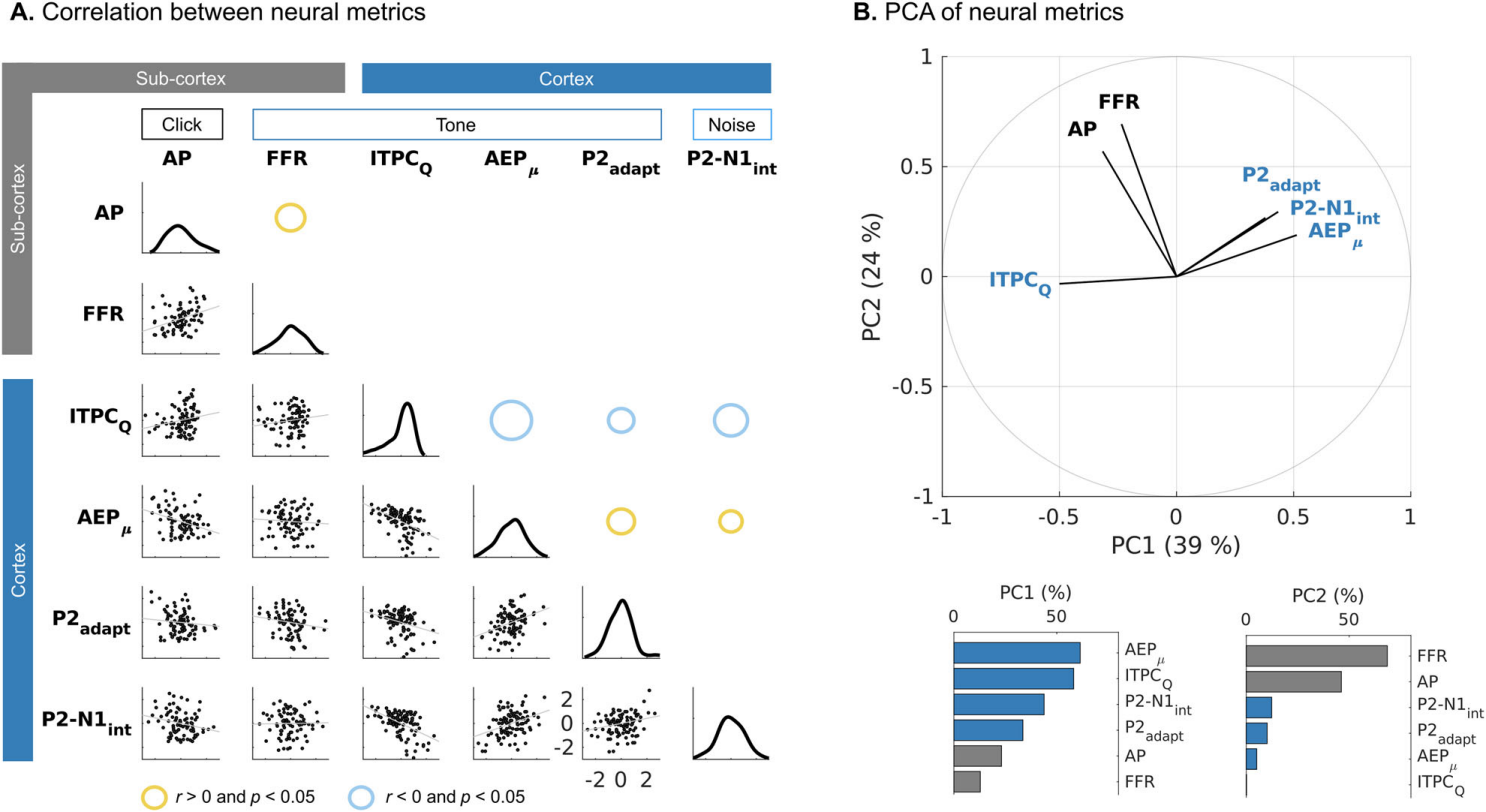
Different trajectories of individual age effects in central and peripheral auditory processing. ***A***, Peripheral and central neural metrics are intracorrelated, but not intercorrelated. Circles represent significant positive (yellow) or negative (blue) correlations after correction for multiple comparisons. AP, auditory nerve action potential component of the ABR wave I (Fig. 2). FFR, brainstem FFRs to tone carriers (Fig. 3). ITPC_Q_, ratio of spectral harmonics describing the periodicity of cortical responses following 2 Hz tone sequences (Fig. 6*D*,*E*). AEPm, mean amplitude of cortical tone-evoked AEPs (Fig. 6*B*). P2_adapt_, adaption in P2 peak amplitudes with tone repetition (Fig. 6C). P2-N1_int_, transient P2-N1 peak differences across repetition rates in noise-evoked AEPs (Fig. 5*E*,*F*). ***B***, PCA of electrophysiological measures showing age-related effects. Peripheral (gray) and central (blue) measures represent different directions of variability in aging humans. Bar and line sizes represent relative explained variance.

A main question was to investigate whether measures of CND in aging humans with normal hearing would predict concomitant age-related changes in cortical sound-evoked responses. Figure 7*A* shows the matrix of pairwise correlations between the main electrophysiological neural metrics from the cochlear nerve (AP amplitudes), the auditory brainstem-midbrain (FFR_SNR_), and the auditory cortex (ITPC_Q_, AEP_μ_, P2_adapt_, P2-N1_int_). We included only metrics that showed significant age-related effects after accounting for threshold differences. As mentioned above, the AP and FFR_SNR_, as complementary metrics of CND, were significantly correlated (*ρ* *=* 0.377, *p* *=* 0.013, corrected). The different cortical response metrics were also mutually correlated. ITPC_Q_ was negatively correlated with AEP_μ_ (*ρ* = −0.530, *p* < 0.001), P2_adapt_ (*ρ* *=* −0.330, *p* *=* 0.010), and P2-N1_int_ (*ρ* *=* −0.435, *p* *<* 0.001) and AEP_μ_ was positively correlated with P2_adapt_ (*ρ* *=* 0.345, *p* 0.005) and P2-N1_int_ (*ρ* *=* 0.294, *p* *=* 0.047). However, no significant correlations were observed between peripheral and central neural metrics, even for measures derived from the same stimulation. This was the case for both the AP (ITPC_Q_: *ρ* *=* 0.265, *p* *=* 0.213; AEP_μ_: *ρ* *=* −0.304, *p* *=* 0.071; P2_adapt_: *ρ* *=* −0.126, *p* *=* 1; P2-N1_int_: *ρ* *=* −0.222, *p* *=* 0.679) and the FFR (ITPC_Q_: *ρ* *=* 0.176, *p* *=* 1; AEP_μ_: *ρ* *=* −0.099, *p* *=* 1; P2_adapt_: *ρ* *=* −0.243, *p* *=* 0.376; P2-N1_int_: *ρ* *=* 0.007, *p* *=* 1). To visualize the covariance structure, Figure 7*B* shows the first two principal components of a PCA of the metrics. Notably, peripheral and central readouts load separately on the two orthogonal dimensions. Cortical readouts (ITPC_Q_, AEP_μ_, P2-N1_int_, P2_adapt_) load mostly on the first component (PC1) and peripheral ones (AP, FFR) on the second component (PC2). Interestingly, PC1 scores correlated significantly with age (*ρ* = −0.659, *p* < 0.001), while PC2 did not (*ρ* *=* −0.156, *p* *=* 0.113).

## Discussion

In an aging human cohort, we observed reduced suprathreshold FFRs and ABR AP amplitudes consistent with age-related CND despite clinically normal hearing thresholds. Perhaps surprisingly, these physiological measures of cochlear neural health did not associate significantly with concurrent age-related changes in cortical responses evoked by the same sound stimuli. This suggests different trajectories of age-related effects on responses to simple tone or noise stimuli in the peripheral versus the central auditory system.

To assess cochlear neural health noninvasively in aging humans, we recorded ABRs, FFRs, and MEMRs as complementary readouts. Their intercorrelation is consistent with CND being a common driver in their reduction, but the metrics may nonetheless be differently sensitive to CND (Bharadwaj et al., 2019). For ABRs recorded in the ear canal, we observed age-related reductions in AP, but not SP amplitudes, consistent with previous reports (Grose et al., 2019; Johannesen et al., 2019; Carcagno and Plack, 2020) and with age-related loss of cochlear nerve fibers. Yet, as a biomarker for CND, the click ABR is complicated by concurrent age-related loss of hair cells. Although participants had normal thresholds at low frequencies, high-frequency thresholds were elevated in the older group (Fig. 1*A*). This decline in hearing sensitivity above audiometric frequencies (i.e., >8 kHz) is a common pattern—and confound—in human aging studies. High-frequency threshold elevations suggest basal OHC loss, effectively reducing the contribution of basal cochlear nerve fibers to the summed broadband response that the click elicits (Don and Eggermont, 1978). To eliminate this basal contribution, Carcagno and Plack (2020) used a high-frequency masking noise, which in turn removed any age-related reduction in click-evoked ABR wave I responses. However, high-frequency masking is also likely to mask the effects of neural degeneration known from human histopathology to occur throughout the cochlear spiral, including basal regions (Wu et al., 2019).

As an alternative, the FFR has been proposed to be minimally affected by basal OHC loss (Märcher-Rørsted et al., 2022) and therefore less biased by high-frequency threshold elevations. The reduction in FFR steady-state activity has been suggested to index cochlear nerve loss although originating from the auditory brainstem (Märcher-Rørsted et al., 2022; Temboury-Gutierrez et al., 2024). With sustained stimulation at lower frequencies (<1,000 Hz), cochlear nerve fibers converging at their target synapses in the lower brainstem produce enhanced synchronization in their targets in the cochlear nucleus relative to the individual contributing fibers (Joris et al., 1994) and the synchronization strength at the postsynaptic output scales with the number of converging inputs (Xu-Friedman and Regehr, 2005). The effects of cochlear nerve loss may therefore be more pronounced in the postsynaptic activity. This may also explain why the steady-state activity measured by the FFR and the transient activity represented by wave V of the ABR show different age effects despite originating from overlapping circuits in the auditory brainstem and midbrain. Plasticity in these central areas may recover transient activity by upregulating overall firing rate, but without recovering the loss of precise temporal phase-locking caused by deafferentation (Chambers et al., 2016; Möhrle et al., 2016).

Notably, peripheral measures of CND were not significantly correlated with age-related changes in cortical AEPs. We observed a general enhancement of cortical transient responses, most salient at long ISIs. Cortical hyperresponsivity has previously been reported in humans with hearing loss (Alain et al., 2014; Millman et al., 2017; Fuglsang et al., 2020) and is often observed in animal models of hearing loss (Popelár̆ et al., 1987; Sun et al., 2012; Salvi et al., 2017) or CND (Chambers et al., 2016; Asokan et al., 2018; Resnik and Polley, 2021). Chambers et al. (2016) used a mouse model of extensive cochlear denervation to show that response amplitudes to suprathreshold transient stimuli were greatly reduced at the level of the cochlear nerve (ABR wave I) but gradually restored in the auditory midbrain and even enhanced pretreatment in auditory cortex (multi-unit activity). This pattern is consistent with our current results in aging humans, showing reduced APs from the cochlear nerve, unchanged ABR wave V (but reduced FFRs) from the auditory brainstem/midbrain, and amplified cortical AEPs. However, cortical hyperactivity is also a common phenotype in aging reported across sensory cortices (Schmolesky et al., 2000; Yu et al., 2006), and the observation of hyperactivity in older humans does not, by itself, guarantee a peripheral origin of effects.

Rather than a uniform response gain in cortex, we observed salient age-related changes in the morphology of transient AEPs evoked by repeated sounds. The P2 reduction and the emergence of a broad negativity at lower ISIs (Fig. 6*A*) have previously been observed in young adults (Bertoli and Probst, 2005; Sussman et al., 2008; Costa-Faidella et al., 2011; Holt and Özdamar, 2016) and could represent a transition toward steady-state activity following the sequence rather than individual onsets. However, it is unclear why the decrease in ISI affects specifically the P2 component and why aging causes enhanced and persistent P2s to repetitive stimuli (Pfefferbaum et al., 1980; Anderer et al., 1996; Amenedo and Díaz, 1998; Fabiani et al., 2006). The P2 component matures early in life and has been proposed to reflect nonlemniscal outputs from the reticular activation system (RAS; Ponton et al., 2000). Persistent P2s evoked by repeated sound onsets could indicate an age-related decline in the RAS-mediated ability to automatically disengage attention from monotonic stimuli (García-Larrea et al., 1992; Crowley and Colrain, 2004).

Hyperactivity or reduced suppression of successive signals can also arise with age-related declines in inhibitory neurotransmission at the cortical level (Wang et al., 2000, 2002; Caspary et al., 2008; Kamal et al., 2013). GABA levels decline with age in auditory cortex (Ling et al., 2005; Caspary et al., 2013; Ouellet and de Villers-Sidani, 2014; Lalwani et al., 2019; Rogalla and Hildebrandt, 2020) and hearing loss may reduce them further (Martin del Campo et al., 2012; Gao et al., 2015; Harris et al., 2022), but declines occur throughout the aging brain (Gao et al., 2013; Porges et al., 2017; Zuppichini et al., 2024). GABAergic neurons exert high metabolic demands and may therefore be particularly susceptible to aging (Kann et al., 2014), and reduced GABA signaling can alter spectrotemporal coding in several aspects (Caspary et al., 2008; Ibrahim and Llano, 2019). In old monkeys, Ng and Recanzone (2018) observed local hyperactivity and spatially redundant activity across cortical areas which could both contribute to hyperresponsiveness observed in far-field EEG. In aged rats, de Villers-Sidani et al. (2010) reported numerous temporal coding deficits associated with decreases in parvalbumin-expressing interneurons in auditory cortex, including reduced adaptive suppression of repeated sounds, and, with that, a poorer behavioral ability to discriminate deviant stimuli. Additionally, functional deficits and neural loss could be reversed by behavioral training. This was suggested to indicate that peripheral inputs remain sufficiently intact for recovery and that functional changes reflect primary aging rather than secondary effects of peripheral denervation (de Villers-Sidani et al., 2010). The existence of such plastic cortical aging might explain the poor correlation between central and peripheral measures observed here in humans.

However, the lack of linear correlation between central and peripheral measures does not, by itself, imply that the observed cortical changes are not causally linked to peripheral decline. Age-related deafferentation could introduce non-monotonic dynamics in the transformations between the auditory periphery and cortex that are not captured by linear correlation. Additionally, if age-related changes can be partly reversed through training (de Villers-Sidani et al., 2010; Mishra et al., 2014), cortical consequences of peripheral deafferentation may evolve differently depending on auditory experience. Nonetheless, the lack of correlation is noticeable, given that we measured cortical AEPs elicited by simple stimuli during passive stimulation and both peripheral and central responses were derived from the same stimulation. Late AEPs, evoked by similar simple stimuli, are used in clinical hearing assessment due to their large amplitudes compared with earlier potentials (Cone-Wesson and Wunderlich, 2003; Kim, 2015). Our present data indicate that a significant portion of the individual variability in cortical AEPs in aging humans relates to cortical aging that may progress in parallel with peripheral decline.

Different aging trajectories in the central and peripheral auditory system may also help to explain mixed results regarding the perceptual consequences of CND in aging humans (Johannesen et al., 2019; Shehorn et al., 2020). Suppression deficits or reduced capacity to disengage attention from irrelevant stimuli are well-known behavioral effects of aging (Hasher and Zacks, 1988; Gazzaley et al., 2005) linked to declines in cortical inhibition (Hermans et al., 2018). Persistent neural responses to redundant information, as observed here, may compromise the discrimination of novel stimuli and increase susceptibility to distraction at the behavioral level (de Villers-Sidani et al., 2010; Mishra et al., 2014). Although domain-general, such deficits may critically impact the performance of complex auditory tasks, such as discrimination of masked signals (Tun et al., 2002; Humes et al., 2012). Compared with young peers, older individuals with similar hearing thresholds vary greatly in performance on complex sound perception tasks (Billings et al., 2015; Varnet et al., 2021), and a considerable portion of this variability may relate to declines in cortical inhibition accompanying brain aging (Harris et al., 2022). In aging humans, Gómez-Álvarez et al. (2023) reported that performance on the visual Stroop task, assessing inhibitory control, predicted speech-in-noise task performance well while physiological measures of CND (ABR wave I growth functions) did not. CND may still contribute to the variability observed in speech perception tasks (Grant et al., 2020; McHaney et al., 2024; Garrett et al., 2025), but the contribution may be small in subjects with normal hearing thresholds (Oxenham, 2016; Johannesen et al., 2019). However, it should be noted that cochlear nerve degeneration is typically highly correlated with simultaneous loss of OHCs in normal aging humans (Wu et al., 2021) and the degree of CND may be less extensive in older listeners with normal hearing. CND may thus still be an important contributor to the correlation between word-recognition scores and audiometric thresholds in listeners with hearing loss while effects of CND may be outweighed by primary aging factors in the absence of overt hearing loss. Compared with peripheral assays, cortical AEPs may better predict behavioral task performance (Billings and Madsen, 2018) but the predicted variance may not necessarily reflect hearing status to any strong degree.

In conclusion, complementary CND measures indicated peripheral neural decline in normal hearing aging listeners, but these were not significantly correlated with cortical hyperactivity or suppression deficits often associated with age-related decline in hearing. While the maintained wave V response amplitudes in the aging auditory midbrain may represent a gain-control adaptation, cortical hyperactivity does not necessarily only represent a compensation of reduced peripheral input. Instead, age-related alterations in cortical sound-evoked activity may be significantly impacted by intrinsic brain plasticity that progress alongside peripheral deafferentation. More direct measures of cortical inhibitory neurotransmission may more clearly disentangle the relative contributions of central inhibitory decline and peripheral degeneration.

## Data Availability

The source data are provided in Dataset S1.
